# Functional characterization of EtAMA3 in *Eimeria tenella* and immunogenicity of its knockout line

**DOI:** 10.1186/s13071-026-07618-4

**Published:** 2026-08-19

**Authors:** Shiqi Du, Ying Pang, Rongqing Dong, Xinming Tang, Changbo Ou, Hongbin Si, Xingju Song, Dandan Hu

**Affiliations:** 1 Guangxi Key Laboratory of Animal Breeding, Disease Control and Prevention, College of Animal Science and Technology, Guangxi University, Nanning, 530004 China; 2 Key Laboratory of Animal Biosafety Risk Prevention and Control (North) and Key Laboratory of Veterinary Biological Products and Chemical Drugs of MARA, Institute of Animal Science, Chinese Academy of Agricultural Sciences, Beijing, 100193 China

**Keywords:** *Eimeria*, Apical membrane antigen, CRISPR-Cas9, Gene knockout, Vaccine

## Abstract

**Background:**

Avian coccidiosis, predominantly caused by *Eimeria* species, imposes severe economic burdens on the global poultry sector. EtAMA3 is an apical membrane antigen specific to *Eimeria tenella* sporozoites, yet its functions in parasite invasion, intracellular development, and utility as a target for rationally attenuated vaccine design remain poorly characterized.

**Methods:**

In this study, we utilized targeted gene knockout and overexpression to dissect the biological functions of EtAMA3 during *E. tenella* invasion and endogenous development.

**Results:**

Our results show that EtAMA3 localizes primarily to the sporozoite apical pole. EtAMA3 deletion only moderately attenuates in vitro sporozoite invasion but severely disrupts cecal schizont formation, drastically curtails oocyst excretion, and mitigates clinical disease and cecal pathology. Importantly, the knockout strain retains robust immunoprotective capacity at standard immunization dosages. Transcriptomic profiling of EtAMA3-deficient sporozoites uncovered suppressed transcription of core moving junction (MJ) components RON2, RON4, and RON8, alongside elevated RON2L2 expression. Loss of EtAMA3 also triggered widespread transcriptional reprogramming of ApiAP2 transcription factors and numerous transmembrane protein-coding genes.

**Conclusions:**

These findings demonstrate that EtAMA3 is an important component of the molecular networks governing *E. tenella* invasion and development, and that the EtAMA3 knockout strain combines reduced virulence with preserved immunogenicity, providing an experimental basis for dissecting invasion mechanisms and developing next-generation genetically attenuated live anticoccidial vaccines.

**Graphical Abstract:**

**Figure:**
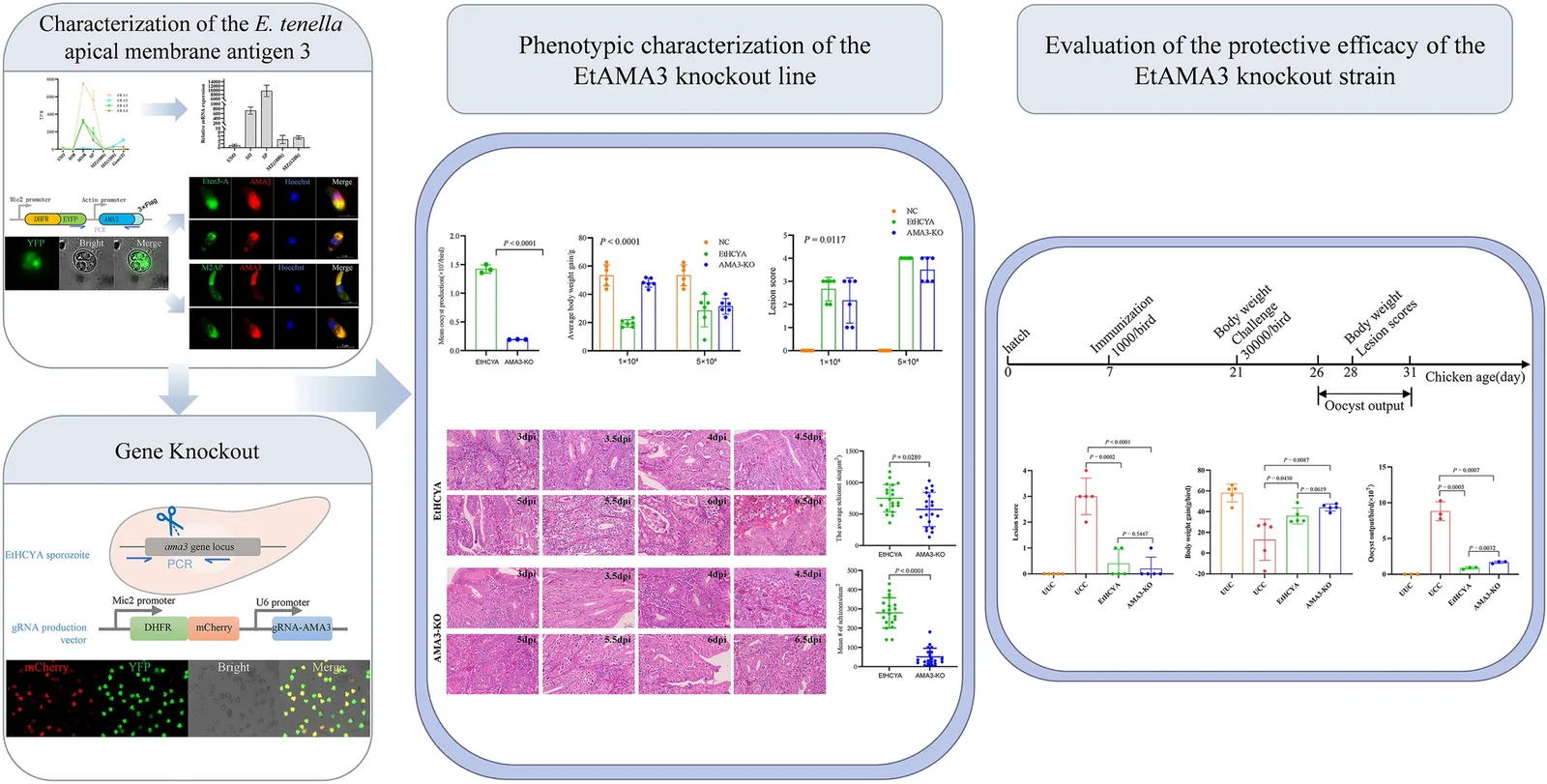

**Supplementary Information:**

The online version contains supplementary material available at https://doi.org/10.1186/s13071-026-07618-4.

## Background

Chicken coccidia are obligate intracellular parasites that develop in the epithelial cells of the small intestine or caeca, where extensive replication causes severe damage to the intestinal mucosa. Infected birds typically present with diarrhea, bloody feces, and anemia, with heavy infections capable of causing acute death [1]. Coccidiosis is therefore a major constraint on intensive poultry production worldwide, resulting in substantial subclinical economic losses due to reduced weight gain and impaired feed conversion, as well as direct losses caused by high mortality in affected flocks. Globally, the annual economic costs of preventing and controlling avian coccidiosis are estimated to surpass £10 billion [2, 3].

Currently, the control of avian coccidiosis relies largely on the prophylactic use of anticoccidial drugs in feed. Nevertheless, prolonged, widespread anticoccidial drug administration has driven pervasive *Eimeria* drug resistance, alongside rising concerns over edible tissue drug residues and environmental pollution. Consequently, vaccination has become an increasingly important strategy for coccidiosis control [4, 5]. All currently licensed commercial coccidiosis vaccines are based on live oocysts, including virulent and attenuated strains. Among them, precocious attenuated vaccines obtained through serial selection of early-maturing lines offer improved safety compared with vaccines based on fully virulent strains. Nonetheless, the time-consuming selection process, together with genetic differences between vaccine strains and field isolates circulating in different regions, can compromise cross-protection and limit the broader application of these vaccines [6, 7]. Thus, the development of novel types of coccidial vaccines remains a high-priority and promising research direction.

Rationally attenuated live vaccines generated by targeted gene deletion have been successfully developed for a variety of bacterial and viral diseases [8], and several promising gene-deleted parasite strains have also emerged as vaccine candidates in parasitology [9–12]. However, progress in this area has been much slower for *Eimeria* spp., largely because little is known about their virulence determinants and, until recently, the previous lack of efficient genetic manipulation tools has severely constrained functional genomic studies [13–15]. As a result, only a few reports [16] have described gene-deleted *Eimeria* strains as potential live attenuated anticoccidial vaccines to date.

During host cell invasion, apicomplexan parasites first secrete effector proteins from micronemes and rhoptries and then assemble a multi-protein “moving junction” (MJ) at the interface between the parasite and the host cell membrane, through which the parasite actively penetrates into the host cell [17, 18]. Previous studies have shown that several MJ components play critical roles in the invasion of *Toxoplasma gondii* and *Plasmodium* spp., and that their disruption can markedly attenuate parasite virulence [19, 20]. In *T. gondii*, at least four interchangeable AMA-RON pairs have been identified (including AMA1-RON2, AMA2-RON2, AMA3-RON2L2, and AMA4-RON2L1) [18], constituting a highly redundant and plastic “invasion module” repertoire [21]. Among these, AMA1-RON2 plays a predominant role, and deletion of AMA1 blocks the majority of parasite invasion events. However, invasion can still occur to a limited extent, likely through compensatory upregulation of AMA2 or engagement of other AMA-based invasion modules [21]. From a vaccine development perspective, such mutants that exhibit only partial impairment of invasion but show markedly reduced virulence without loss of viability display desired characteristics of live attenuated *Eimeria* vaccines.

In contrast, studies on MJ proteins in *Eimeria* spp. remain limited. This study focuses on the moving junction protein AMA3 of *E. tenella*. Using a CRISPR/Cas9-based genome editing system, we functionally characterized EtAMA3 and generated gene knockout parasites to evaluate their potential as gene-deleted attenuated vaccine candidates. Our findings deepen the understanding of the invasion process in *E. tenella* and provide a new conceptual framework for vaccine-based control of coccidiosis.

## Methods

### Ethics statement

All procedures involving animals were reviewed and approved by the Laboratory Animal Administration Committee of Guangxi University and carried out in strict accordance with the institutional guidelines for the care and use of laboratory animals (protocol no. GXU-2024-169) and applicable veterinary guidance. Birds were monitored at least twice daily for health status, and humane endpoints were predefined. At planned endpoints or if humane endpoints were met, chickens were anesthetized with isoflurane until loss of the righting reflex, and then euthanized by cervical dislocation. Death was confirmed by cessation of heartbeat and respiration.

### Animals and parasites

An *E. tenella* Houghton line constitutively expressing Cas9 (EtHCYA), generated and maintained in our laboratory as described previously [13], was used as the parental strain for generation of knockout mutants, whereas the wild-type Houghton strain (gift from Prof. Xun Suo at China Agricultural University) served as the background for EtAMA3 overexpression. Parasites were maintained by serial passage in 1- to 4-week-old Sanhuang chickens. Birds were kept under coccidia-free conditions and provided ad libitum access to water and to feed devoid of coccidiostats and antibiotics. Oocysts were recovered, purified, and induced to sporulate following previously described protocols [22].

### Plasmid construction

The CRISPR/Cas9 knockout plasmid harboring the U6-driven gRNA cassette and the DHFR–mCherry-positive selection cassette was generated as described previously [13], with minor modifications. In brief, single guide RNAs (sgRNAs) targeting EtAMA3 were designed using EuPaGDT, and the original gRNA sequence in the YFP-KO template vector was replaced by seamless cloning (ClonExpress MultiS One Step Cloning Kit, Vazyme, Nanjing). In this construct, the mCherry reporter was fused in-frame to the pyrimethamine resistance gene TgDHFR-ts-m2m3 (DHFR), and the fusion cassette was placed under the control of the EtMic2 promoter, whereas the gRNA expression was driven by the *E. tenella* U6 promoter.

For overexpression of EtAMA3, the coding sequence of EtAMA3 was amplified from cDNA of *E. tenella* sporozoites and cloned into a Flag-tagged expression vector. The AMA3-Flag fusion was expressed under the regulation of the 5′- and 3′-UTRs of *EtActin*, and a separate DHFR–EYFP cassette, used for drug selection and fluorescence screening, was driven by the EtMic2 promoter. All PCR reactions were carried out with Phanta Max Master Mix (Vazyme, Nanjing), and the primers used in this study are listed in Supplementary material 1.

### Generation of EtAMA3 knockout and overexpression parasite lines

Genetic manipulation of *Eimeria* spp. was performed with minor adaptations from our previously established protocol [23]. Sporocysts of EtHCYA or wild type (WT) were obtained from freshly purified oocysts after disruption with glass beads and subsequent separation on a 50% Percoll (Solarbio, Beijing, China) density gradient. Sporocysts were then excysted in bile–trypsin buffer at 42 °C for 60 min to release sporozoites.

Knockout and overexpression plasmids were linearized with SnaBⅠ at 37 °C for 60 min prior to transfection. Approximately 1 × 10^7^ freshly excysted sporozoites were resuspended in cytomix buffer (final volume 100 μL) and electroporated with 20 μg of each linearized construct using a Nucleofector 2B device (Lonza) with program U-033. Immediately after electroporation, sporozoites were administered via the cloaca to five 2-week-old chickens. Drug-resistant transgenic parasites were enriched over several passages under pyrimethamine pressure (150 ppm in feed; Aladdin, Shanghai, China) and further purified based on fluorescence by flow cytometry (FACSAria II, BD, USA).

### Indirect immunofluorescence assay (IFA)

IFA staining was carried out using the EtAMA3 overexpression line to determine the subcellular distribution. Sporozoites were prepared as described above, and freshly released extracellular parasites were allowed to adhere to poly-L-lysine-coated glass slides. Then, the samples were fixed; the EYFP fluorescence was quenched by incubating the samples in methanol at − 20 °C for 2 h. Cells were then permeabilized with 0.25% Triton X-100 in PBS for 8 min and blocked with 3% bovine serum albumin (BSA) for 30 min at room temperature. After blocking, parasites were incubated for 1 h at 37 °C with mouse anti-Flag antibody (1:500, Abmart, Shanghai, China), rabbit polyclonal anti-EtM2AP antibody (microneme marker), and rabbit polyclonal anti-eten5-A antibody (rhoptry marker). Following several washes in PBS, slides were incubated with Cy3-conjugated goat anti-mouse/rabbit IgG (1:100, Huaxinbio, Beijing, China) for 1 h at 37 °C, and nuclei were counterstained with Hoechst 33258 (1:100, Sigma, St. Louis, MO, USA). Fluorescence images were acquired using a Zeiss fluorescence microscope (Zeiss, Germany).

### Kinetics and quantification of oocyst shedding

For each parasite line, five 7-day-old Sanhuang broiler chicks were orally inoculated with 1000 freshly sporulated oocysts per bird. Fecal samples were collected daily from 5 to 12 days post-infection (dpi), and oocyst shedding was quantified using an advanced McMaster counting chamber to generate oocyst excretion curves. In addition, all feces produced between 5 and 12 dpi were pooled for each group to determine the total oocyst output. Each assay was performed in at least three independent experiments.

### Virulence

To compare pathogenicity, groups of six 7-day-old Sanhuang chicks were infected with either 1 × 10^4^ or 5 × 10^4^ freshly sporulated oocysts per bird of the EtAMA3-KO strain or its parental EtHCYA line. Body weights were recorded on the day of infection (day 0) and at 10 dpi to calculate average weight gain. For lesion scoring, birds were euthanized at 7 dpi, and ceca were removed and scored for gross lesions according to previously published criteria [24]. Total oocyst output was also determined for these groups as described above.

### Endogenous development analysis

To examine endogenous development, 7-day-old chicks were orally challenged with 1 × 10^4^ sporulated oocysts of either EtHCYA or the EtAMA3-KO strain. Ceca were collected from infected birds at 0.5-day intervals between 3 and 6.5 dpi. Tissue samples were fixed, processed for hematoxylin and eosin (H&E) staining, and examined microscopically. For each time point, the mean number of schizonts of intestinal tissue and the average schizont size were determined by analyzing at least 20 randomly selected microscopic fields or individual schizonts.

### In vitro sporozoite invasion assay

For invasion assays, 5 × 10^5^ freshly excysted sporozoites of EtHCYA or the EtAMA3-KO strain were added to confluent DF-1 cell monolayers in 12-well plates and incubated for 2, 4, or 8 h. After incubation, non-invaded parasites were removed by washing the monolayers three times with PBS, and all remaining extracellular sporozoites in each well were counted using a hemocytometer. Invasion efficiency was calculated as follows: The invasion rates = (total number of inoculated sporozoites−uninvaded sporozoites) / total number of inoculated sporozoites × 100%. These experiments were repeated three times.

This invasion quantification method relies on indirect counting of extracellular uninvaded sporozoites and carries inherent limitations. To minimize these confounding factors, we implemented strict standardized experimental controls: uniform washing cycles and identical PBS buffer volume across all groups, exclusive use of freshly excysted viable sporozoites, three fully independent biological replicates with identical incubation conditions, and highly consistent invasion rate trends observed across replicates to reduce random methodological bias.

### Immunoprotection assays

Forty 7-day-old Sanhuang chicks were randomly allocated into four groups (*n* = 10 per group): an unimmunized-unchallenged control (UUC), an unimmunized-challenged control (UCC), an EtHCYA-immunized group, and an EtAMA3-KO-immunized group. Birds in the immunized groups received 1,000 freshly sporulated oocysts of either EtHCYA or the EtAMA3-KO strain by oral gavage, whereas the UUC and UCC groups were left unimmunized. At 14 dpi, all birds except those in the UUC group were orally challenged with 3 × 10^4^ sporulated oocysts of the parental EtHCYA line. Five days after challenge, cecal lesions were scored, and total oocyst output and average body weight gain were determined as described above. The anticoccidial index (ACI) was then calculated according to previously published criteria [25].

### RNA-Seq and data analysis

Total RNA was extracted from sporozoites of the EtHCYA and EtAMA3-KO strains using TRIzol reagent, and residual genomic DNA was removed by treatment with DNase I (Tiangen Biotech Co., Ltd, Beijing, China). RNA purity, concentration, and integrity were evaluated with a NanoPhotometer^®^ spectrophotometer (IMPLEN, CA, USA), a Qubit^®^ 2.0 Fluorometer using the Qubit^®^ RNA Assay Kit (Life Technologies, CA, USA), and an Agilent 2100 Bioanalyzer with the RNA Nano 6000 Assay Kit (Agilent Technologies, CA, USA), respectively. RNA-Seq libraries were prepared from high-quality RNA using the TruSeq™ RNA Sample Preparation Kit (Illumina, CA, USA) following the manufacturer’s protocol, and sequenced on an Illumina NovaSeq 6000 platform to generate 150-bp paired-end reads (Novogene, Beijing, China). Three independent biological replicates were performed.

Adapter-trimmed, high-quality paired-end reads were mapped to the *E. tenella* reference genome [26] (ToxoDB, release 68) using HISAT2 v2.2.1 [27]. The resulting SAM files were converted to BAM format, sorted, and indexed with SAMtools [28]. Gene-level read counts were obtained using HTSeq-count v0.13.5 [29]. Differential gene expression between EtHCYA and EtAMA3-KO sporozoites was analyzed in R using the DESeq2 package [30], and Gene Ontology (GO) and Kyoto Encyclopedia of Genes and Genomes (KEGG) pathway enrichment analyses were carried out with clusterProfiler v4.0.5 [31]. Genes with an absolute log_2_ fold change ≥ 1 and an adjusted *P* value < 0.01 were considered significantly differentially expressed. Transcripts per million (TPM) were calculated for each gene and used to generate clustered heatmaps.

### Reverse transcription quantitative real-time PCR

Total RNA was extracted from sporozoites of the EtHCYA and EtAMA3-KO strains using TRIzol reagent, and residual genomic DNA was removed by treatment with DNase I (Tiangen Biotech Co., Ltd, Beijing, China). Four core moving junction-related genes (RON2, RON4, RON8, RON2L2) and four representative differentially expressed ApiAP2 transcription factors were selected for validation. Reverse transcription was performed to generate cDNA, PCR reactions were conducted on the Roche LightCycler^®^ 480 system using the 2 × M5 HiPer SYBR Premix EsTaq (Mei5 Biotechnology, Beijing). Reactions were carried out in triplicate for each sample. The primers used are listed in Supplementary material 1.The expression of each gene was normalized to the reference gene glyceraldehyde 3-phosphate dehydrogenase (GAPDH).

### Statistical analysis

Unpaired multiple *t*-tests were used to analyze total oocyst output, body weight gain, lesion scores, and sporulation rate. Two-way ANOVA was used for the invasion rate, oocyst output curve, and virulence studies. All quantitative data are displayed as mean ± standard deviation (SD); error bars denote SD. Statistical analyses were performed in GraphPad Prism (v 9) and R (v 4.2). Normality and homogeneity of variance were assessed (e.g., Shapiro–Wilk and Levene’s tests); when assumptions were not met, data were log-transformed or analyzed using appropriate non-parametric tests. Exact n values and the number of independent biological replicates are stated in the corresponding Methods subsections and figure legends. Sample sizes were selected based on prior experience with *E. tenella* infection models and published studies, and no a priori power calculation was performed. Where applicable, birds were allocated to experimental groups using simple randomization (random number generator), all cecal lesion scoring and histological schizont quantification were performed by independent investigators blinded to group allocation. In addition to significance testing via t-tests and ANOVA, standardized effect sizes and 95% confidence intervals (CIs) were calculated for all intergroup comparisons to quantify the magnitude of phenotypic differences, and these metrics are included in Supplementary material 3.

## Results

### Characterization of the *E. tenella* apical membrane antigen 3 (EtAMA3)

**Fig. 1:**
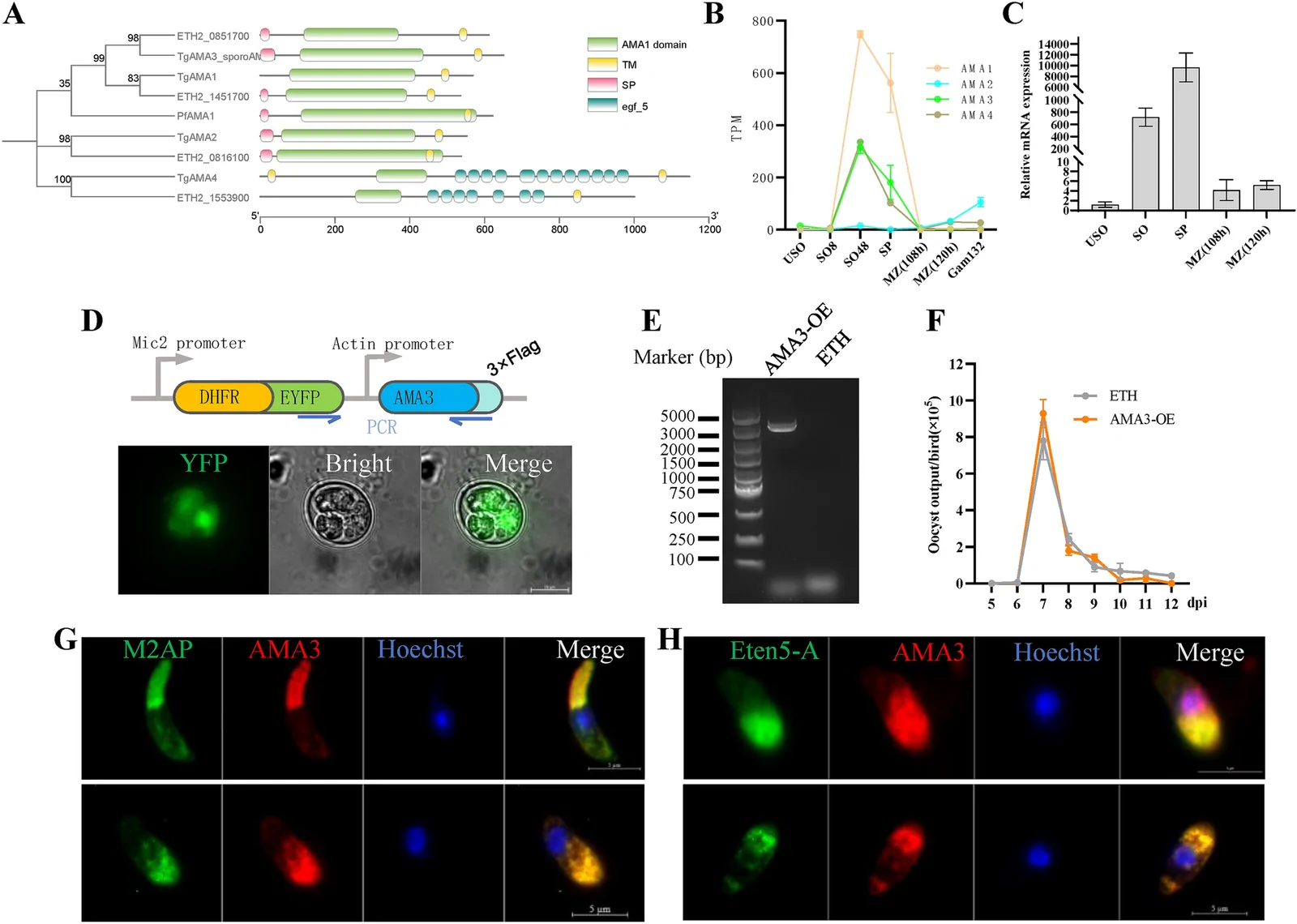
Expression pattern and subcellular localization of EtAMA3. **A** Phylogenetic analysis and domain architecture of AMA proteins. A maximum-likelihood tree was constructed with 1000 bootstrap replicates based on AMA amino acid sequences, and conserved domains were annotated using the InterPro database (*P* < 0.01) and depicted as schematic protein diagrams. **B** Stage-specific transcription profiles of the four *E. tenella* AMA genes based on the RNA-Seq dataset of Chen et al. [34]. USO, unsporulated oocysts; SO8, partially sporulated oocysts; SO, fully sporulated oocysts; SP, sporozoites; MZ(108 h) and MZ(120 h), merozoites collected at 108 h and 120 h post-infection, respectively; GAM132, gametocytes collected at 132 h post-infection. **C** qPCR validation of EtAMA3 transcript levels at different developmental stages. **D** Schematic representation of the EtAMA3 overexpression construct and fluorescent AMA3-OE oocysts. **E** PCR confirmation of the EtAMA3-OE line. **F** Oocyst shedding kinetics of the EtAMA3-OE line compared with the parental wild-type strain. **G**, **H** Indirect immunofluorescence assay showing the subcellular localization of EtAMA3 in sporozoites. EYFP fluorescence in the EtAMA3-OE line was quenched by methanol treatment, and parasites were stained with mouse anti-Flag monoclonal antibody (red) together with rabbit anti-M2AP (microneme marker) or rabbit anti-Eten5-A (rhoptry marker). Nuclei were counterstained with Hoechst 33,258. Scale bar = 5 μm. Error bars represent standard deviation (SD)

Wang et al. (2021) [32] analyzed the apical membrane antigen (AMA) family of *E. tenella* and, based on conserved AMA domains and sequence similarity to *Toxoplasma gondii* AMAs, classified them into four members, EtAMA1–4. Consistent with that work, our domain prediction showed that EtAMA1–3 share a typical architecture consisting of an N-terminal signal peptide, a central AMA conserved domain, and a C-terminal transmembrane segment, whereas EtAMA4 lacks a signal peptide and is instead characterized by multiple epidermal growth factor (EGF)-like domains (Fig. 1A).

Using publicly available *E. tenella* transcriptome data across the parasite life cycle [33], we found that EtAMA1, EtAMA3, and EtAMA4 are highly transcribed in sporulated oocysts and sporozoites, with markedly lower expression at other stages, indicating that they are sporozoite-enriched or sporozoite-specific genes (Fig. 1B). In contrast, EtAMA2 was expressed at relatively low levels overall, but showed a peak in transcript abundance at approximately 132 h post-infection, corresponding to the gametocyte stage (Fig. 1B). Among these family members, EtAMA3 is orthologous to the sporozoite-specific AMA1 (sporoAMA1) of *T. gondii* (Fig. 1A), prompting us to further investigate its role in sporozoite invasion and in parasite development in *E. tenella*.

We first validated the stage-specific expression pattern of EtAMA3 by qPCR. Consistent with the RNA-Seq data, EtAMA3 transcripts were abundant in sporozoites, significantly reduced in sporulated oocysts, and detectable only at very low levels in unsporulated oocysts and merozoites (Fig. 1C). To determine the subcellular localization of EtAMA3 in sporozoites, we generated an overexpression line expressing a C-terminally Flag-tagged EtAMA3 (Fig. 1D, F). Immunofluorescence staining of sporozoites with an anti-Flag antibody, together with the microneme marker EtM2AP and the rhoptry marker Eten5-A, revealed that EtAMA3 localized to the apical region of sporozoites, overlapping with the apical microneme/rhoptry compartment (Fig. 1G, H). These findings are in agreement with those reported previously [33] and support the conclusion that EtAMA3 is a sporozoite-specific apical protein that is likely to participate in the invasion process.

### Generation and verification of the EtAMA3 knockout line

**Fig. 2:**
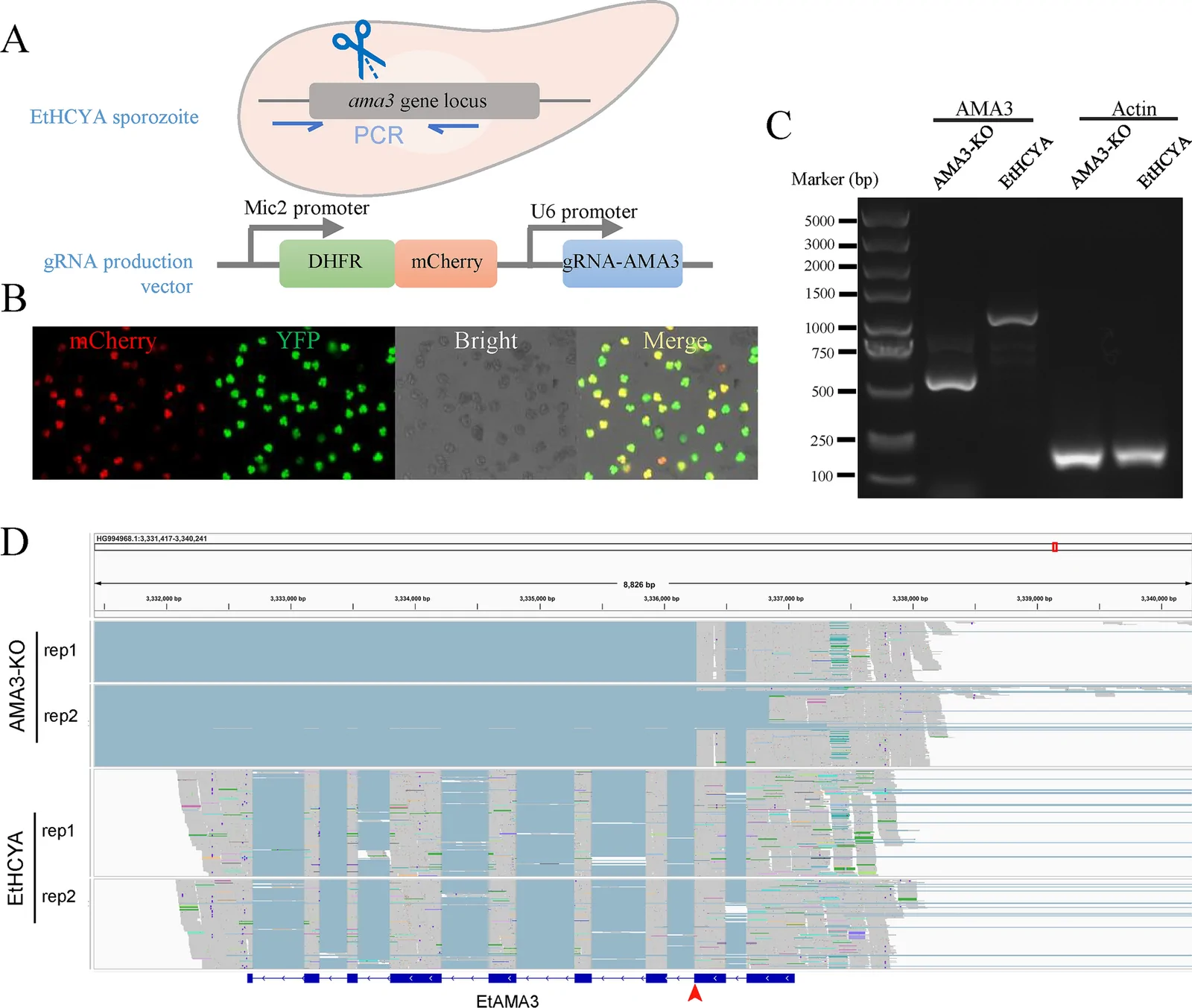
Generation and characterization of the EtAMA3 knockout strain. **A** Schematic representation of the EtAMA3 knockout construct and strategy for disrupting the EtAMA3 locus in the EtHCYA parental line. **B** Fluorescence microscopy of the EtAMA3-KO strain showing mCherry-positive oocysts after drug selection and enrichment. **C** PCR analysis of the EtAMA3 locus in the EtAMA3-KO and EtHCYA strains using primers flanking the gRNA target site, illustrating multiple edited alleles in the knockout population. **D** IGV screenshots of Illumina RNA-Seq reads mapped to the EtAMA3 locus, showing deletions (blue boxes) in different biological replicates of the EtAMA3-KO strain. The gRNA target site is indicated by a red arrowhead

To investigate the contribution of *EtAMA3* to parasite development and sporozoite invasion, we generated an endogenous knockout of the *EtAMA3* locus. A plasmid expressing an *EtAMA3*-specific gRNA together with the DHFR–mCherry selection cassette was constructed and linearized, then introduced into the Cas9-expressing EtHCYA background line by electroporation (Fig. 2A). Following successive rounds of drug selection and fluorescence-activated cell sorting, we obtained a transgenic population in which > 90% of parasites were fluorescent, hereafter referred to as the EtAMA3-KO line (Fig. 2B).

PCR analysis of the *EtAMA3* locus using primers flanking the CRISPR target site revealed a single band of the expected size in the parental EtHCYA strain, whereas multiple bands of smaller sizes were detected in the EtAMA3-KO population (Fig. 2C), consistent with Cas9-induced insertion-deletion (InDel) events around the gRNA target region. To further confirm these editing events at the genomic level, we performed RNA-Seq on EtHCYA and EtAMA3-KO sporozoites and mapped the reads to the *E. tenella* reference genome. Alignment of reads across the *EtAMA3* locus revealed multiple deletions of varying lengths in the vicinity of the gRNA target site in the EtAMA3-KO line (Fig. 2D), thereby confirming successful disruption of *EtAMA3*.

### Phenotypic characterization of the EtAMA3 knockout line

**Fig. 3:**
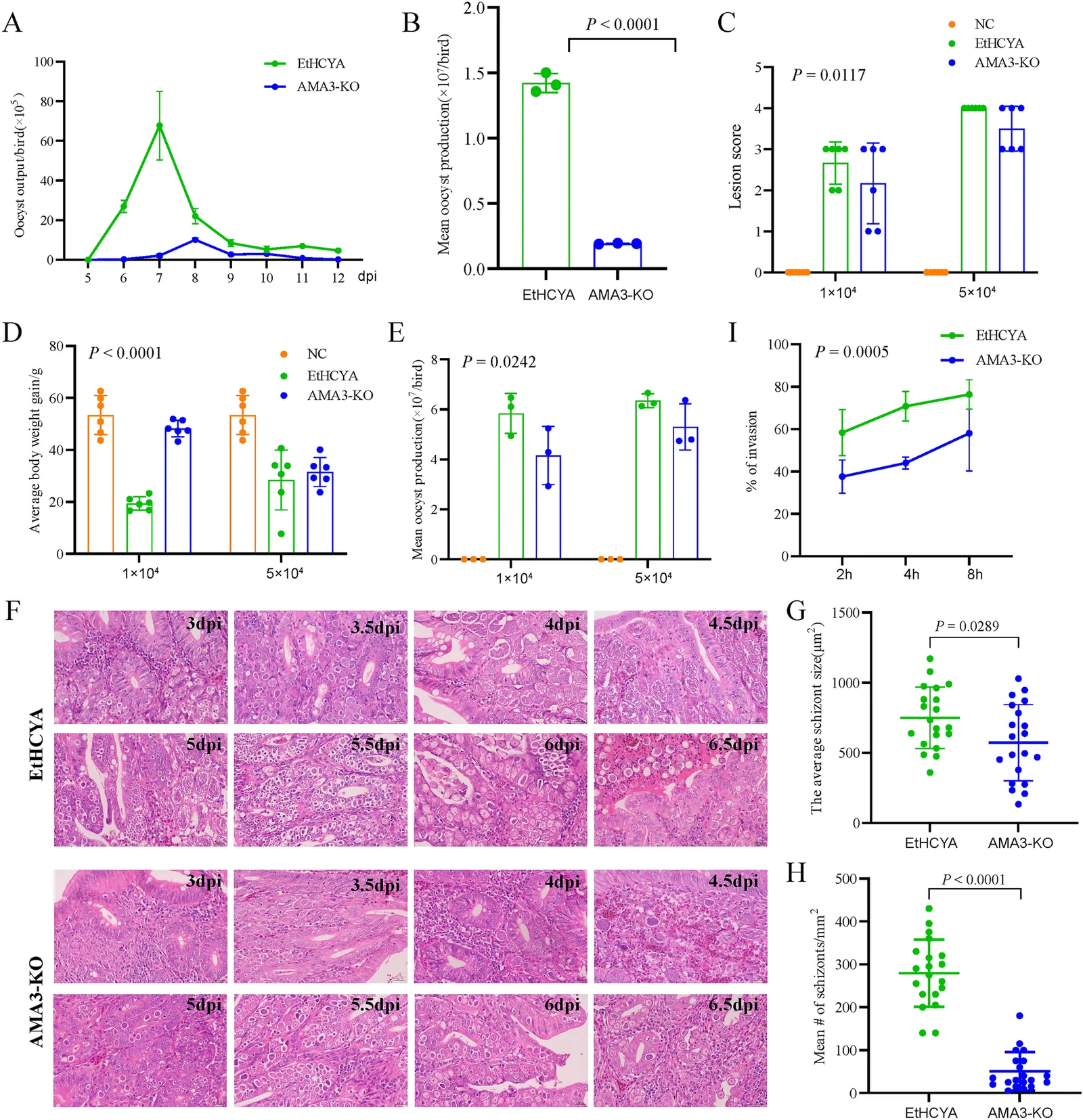
Biological characteristics of the EtAMA3 knockout strain. **A** Oocyst shedding kinetics of the EtAMA3-KO and EtHCYA strains. Chickens (*n* = 6) were orally infected with 1,000 freshly sporulated oocysts/bird, and daily oocyst output was recorded from 5 to 12 dpi. **B** Total oocyst output per bird for the experiment shown in panel **A**. **C**–**E** Comparison of pathogenicity between EtAMA3-KO and EtHCYA strains. Each bird was infected with either 1 × 10^4^ or 5 × 10^4^ freshly sporulated oocysts of EtAMA3-KO or EtHCYA, and cecal lesion scores (**C**), average body weight gain (**D**), and total oocyst output (**E**) were determined at the indicated time points. **F** Representative H&E-stained cecal sections from chickens infected with EtAMA3-KO or EtHCYA strains. **G**, **H** Quantitative analysis of schizogony at 108 h post-infection (4.5 dpi). The average schizont size (**G**) and mean number of schizonts (**H**) were determined from at least 20 randomly selected microscopic fields per group. **I** In vitro invasion efficiency. DF-1 cell monolayers were infected with 5 × 10^5^ sporozoites for 2, 4, or 8 h, after which non-invaded parasites were removed by washing and counted. Lesion scoring and histological counting were conducted by blinded researchers. *n* = 6 chickens per group, three independent biological replicates. Error bars represent standard deviation (SD)

We next examined the biological characteristics of the EtAMA3-KO line. First, oocyst shedding dynamics were assessed by orally inoculating chickens with 1,000 sporulated oocysts per bird and monitoring daily oocyst output. Compared with the parental EtHCYA strain, the EtAMA3-KO line displayed a markedly flattened oocyst output curve (Fig. 3A). EtHCYA showed a sharp peak at 7 dpi, whereas the EtAMA3-KO peak was delayed to 8 dpi and its magnitude was substantially reduced, with the total oocyst output per bird decreased approximately sevenfold (Fig. 3B). No oocysts were detected for either strain at 5 dpi, and the prepatent period did not differ appreciably between the two lines. In contrast, AMA3 overexpression did not significantly alter either the oocyst shedding profile or the total oocyst output compared with the wild-type strain (Fig. 1F).

To evaluate the impact of EtAMA3 disruption on virulence, two infection doses (1 × 10^4^ and 5 × 10^4^ sporulated oocysts per bird) were administered to Sanhuang chickens, and cecal lesions were scored at 7 dpi. Relative to the EtHCYA control, the EtAMA3-KO strain showed a trend toward reduced pathogenicity, but the difference in lesion scores reached statistical significance only at the higher challenge dose of 5 × 10^4^ oocysts (*P* < 0.05, Fig. 3C). In terms of growth performance, birds infected with 1 × 10^4^ oocysts of the EtAMA3-KO line had significantly greater body weight gain than those infected with EtHCYA (*P* < 0.0001), whereas at the higher dose of 5 × 10^4^ oocysts, weight gain did not differ significantly between the two groups (Fig. 3D). Analysis of oocyst shedding under these conditions showed that total oocyst output increased with dose in the EtAMA3-KO group, while remaining at a relatively high and stable level in the EtHCYA-infected group (Fig. 3E). Although oocyst excretion in EtAMA3-KO-infected birds tended to be lower than in EtHCYA-infected birds, these differences did not reach statistical significance. Taken together, these findings suggest that EtAMA3 deletion confers partial attenuation of virulence, but that this effect can be diminished or masked when the infection dose is further increased.

We then compared endogenous development of the EtAMA3-KO and EtHCYA strains in vivo. Ceca were collected from infected chickens between 3 and 7 dpi, processed for H&E staining, and examined histologically. Qualitatively, the developmental stages observed at each time point were similar between the two strains, with no obvious delay in the progression of endogenous stages in the EtAMA3-KO line (Fig. 3F). However, careful microscopic examination suggested that schizonts of the knockout line appeared smaller. We therefore quantitatively measured the average schizont size and the number of schizonts per field at 4.5 dpi. EtAMA3-KO schizonts were significantly smaller than those of EtHCYA (*P* = 0.0289, Fig. 3G), and the number of schizonts in random fields was also significantly reduced (*P* < 0.0001, Fig. 3H). These data indicate that although the timing of developmental progression is broadly comparable between the two lines, the schizogony phase in the EtAMA3-KO strain is compromised, which likely contributes to the reduced oocyst output observed in vivo.

Given that EtAMA3, the ortholog of sporoAMA1 in *T. gondii*, is a sporozoite-specific AMA and that EtAMA3 is highly expressed in *E. tenella* sporozoites, we hypothesized that EtAMA3 might play an important role in sporozoite invasion. To test this, we compared the in vitro invasion efficiency of sporozoites from the EtAMA3-KO and EtHCYA strains. At 2–8 h post-inoculation of DF-1 cell monolayers, the proportion of invaded sporozoites was consistently lower for EtAMA3-KO than for EtHCYA, and overall invasion efficiency was significantly reduced in the knockout line (*P* = 0.005, Fig. 3I). Notably, however, EtAMA3-KO sporozoites retained substantial invasive capacity, with invasion rates decreased by approximately 24–37% relative to EtHCYA but not completely abolished (Fig. 3I). This partial reduction in invasion suggests that additional paralogous genes or other adhesins may partially compensate for the loss of EtAMA3.

### Evaluation of the protective efficacy of the EtAMA3 knockout strain

**Fig. 4:**
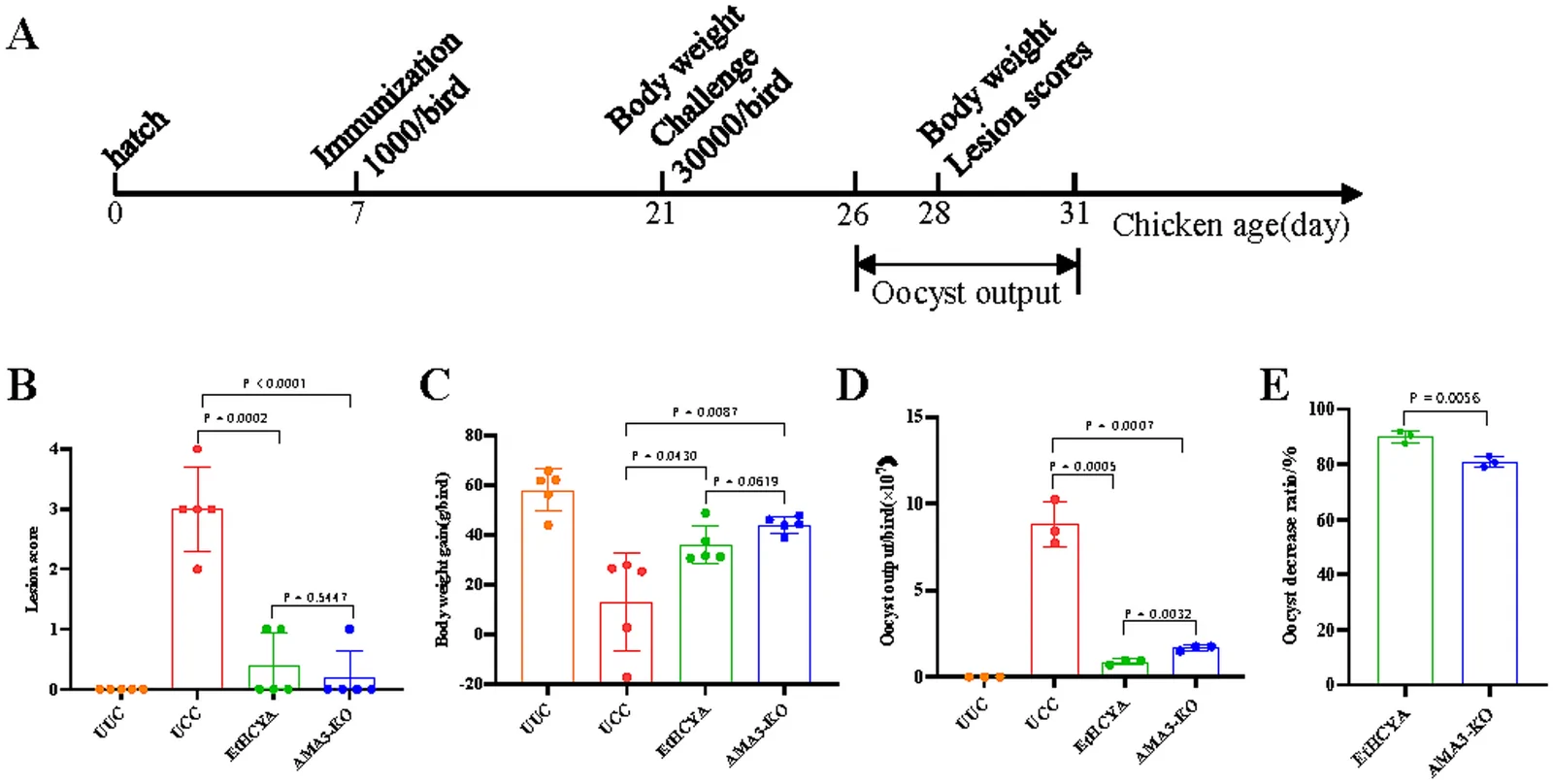
Evaluation of the protective efficacy of the EtAMA3 knockout strain. **A** Schematic overview of the immunization and challenge protocol. Seven-day-old chickens were orally immunized with 1000 sporulated oocysts of either the EtAMA3-KO or EtHCYA strain and challenged at 14 dpi with 3 × 10^4^ sporulated oocysts of the parental EtHCYA line. **B**–**E** Cecal lesion scores (**B**), average body weight gain (**C**), total oocyst output per bird (**D**), and oocyst reduction ratio relative to the unimmunized-challenged control (**E**). *UUC* unimmunized and unchallenged control group, *UCC* unimmunized and challenged control group. Lesion scoring and histological counting were conducted by blinded researchers. *n* = 5 chickens per group, three independent biological replicates. Error bars represent standard deviation (SD)

Because the EtAMA3-KO line exhibited reduced virulence and impaired in vivo development, we next assessed its immunogenicity to determine its potential as a gene deletion live vaccine. Seven-day-old chicks were orally immunized with 1000 freshly sporulated oocysts per bird of either the EtAMA3-KO or EtHCYA strain and challenged 14 days later with 3 × 10^4^ sporulated oocysts of the parent line (Fig. 4A). Cecal lesions were scored 7 days after challenge. Birds immunized with EtAMA3-KO showed the lowest lesion scores (0.2 ± 0.45), which were significantly lower than those of the unimmunized-challenged control group (UCC; 3.8 ± 0.45, *P* < 0.0001). The lesion score in the EtHCYA-immunized group was 1.0 ± 0.71, and the difference between the EtAMA3-KO- and EtHCYA-immunized groups was not statistically significant (Fig. 4B).

As for the average body weight gain, birds in the UCC group exhibited a marked reduction after challenge, whereas immunization with either EtAMA3-KO or EtHCYA largely restored weight gain. Notably, the mean body weight gain in the EtAMA3-KO-immunized group was significantly higher than that in the EtHCYA-immunized group (*P* < 0.05, Fig. 4C), with relative weight gains of 73% and 70%, respectively. In addition, both EtAMA3-KO and EtHCYA immunization significantly reduced total oocyst shedding after challenge compared with the UCC group (Fig. 4D). The reduction in oocyst output reached 81% in the EtAMA3-KO-immunized group and 90% in the EtHCYA-immunized group (Fig. 4E), indicating a slightly lower, but still substantial, suppression of oocyst excretion in the EtAMA3-KO group. Based on these parameters, the anticoccidial indices (ACI) for the EtAMA3-KO- and EtHCYA-immunized groups were calculated to be 169 and 153, respectively. Overall, these data indicate that deletion of EtAMA3 results in only partial attenuation of parasite virulence while preserving, and in some aspects slightly enhancing, the protective efficacy of the parasite, supporting the EtAMA3-KO line as a promising gene deletion live vaccine candidate against chicken coccidiosis.

### EtAMA3 deletion leads to extensive changes in gene expression

**Fig. 5:**
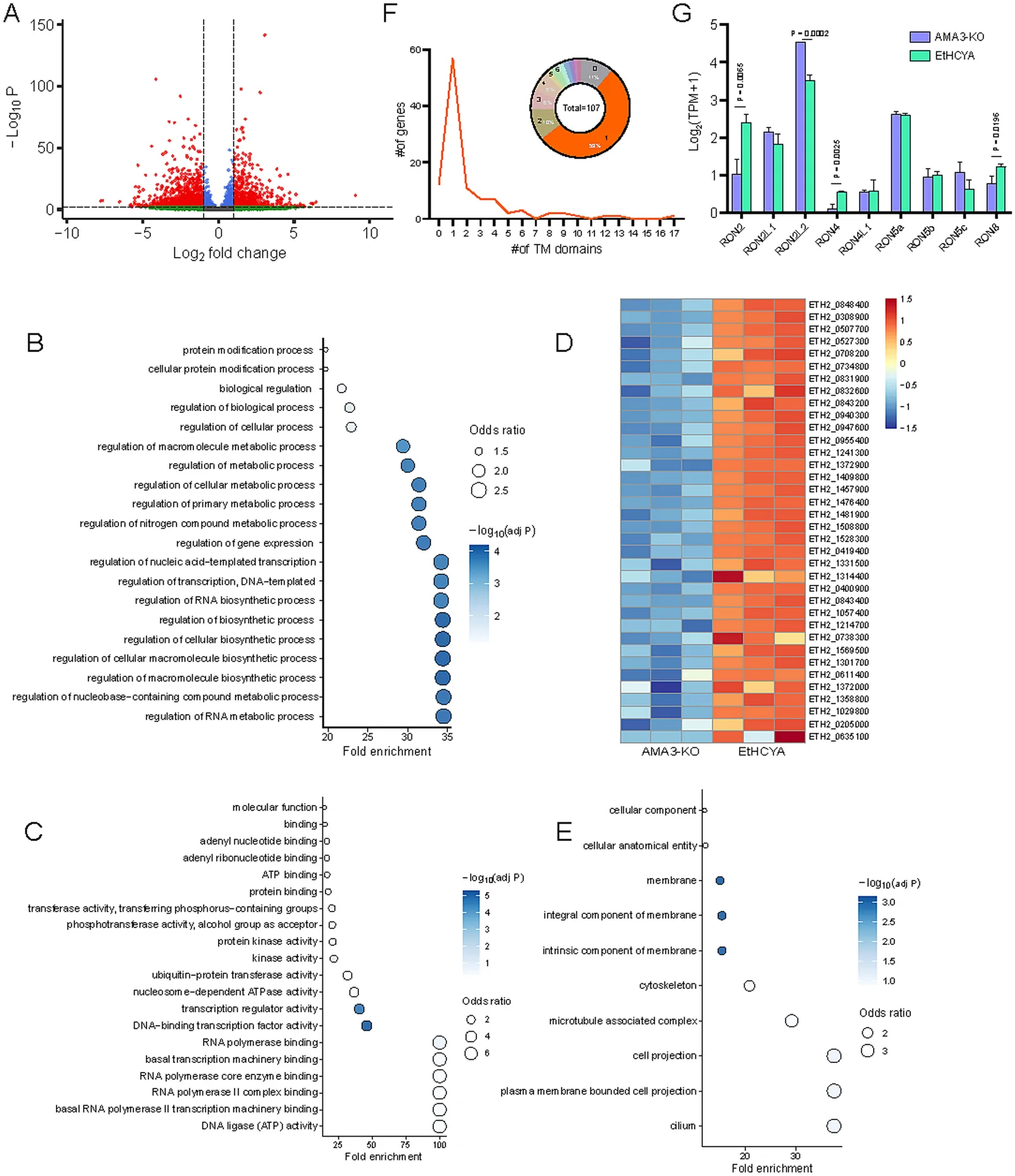
Transcriptomic analysis of the EtAMA3 knockout strain. **A** Volcano plot of DEGs between EtAMA3-KO and EtHCYA sporozoites (adjusted *P* < 0.01, |log_2_FC|≥ 1). Green and red dots indicate significantly downregulated and upregulated genes, respectively. **B**, **C** Gene Ontology (GO) enrichment analysis of downregulated DEGs in the Biological Process (**B**) and Molecular Function (**C**) categories. **D** Heatmap of significantly enriched downregulated genes. Genes annotated as AP2 transcription factors are highlighted in red. **E** GO enrichment analysis of upregulated DEGs in the Cellular Component category. **F** Distribution of predicted transmembrane domains among genes contributing to the significantly enriched CC terms, based on annotations from ToxoDB. **G** Differential expression of RON family genes encoding moving junction components in EtAMA3-KO versus EtHCYA sporozoites. Error bars represent standard deviation (SD)

To further delineate the impact of *EtAMA3* disruption on the parasite, we performed RNA-Seq on sporozoites of the EtAMA3-KO and EtHCYA strains. In total, 1,771 genes were identified as differentially expressed between the two lines (|log_2_ fold change|≥ 1, adjusted *P* < 0.01), including 758 upregulated and 1,013 downregulated genes in the EtAMA3-KO strain (Fig. 5A, Supplementary material 2).

GO enrichment analysis of the downregulated genes revealed significant overrepresentation of terms within the Biological Process (BP) and Molecular Function (MF) categories (adjusted *P* < 0.05, Supplementary material 2), particularly those related to DNA binding, transcription regulation, and regulation of gene expression (Fig. 5B, C). Examination of the genes contributing to these enriched terms identified a core set of 36 DEGs (Fig. 5D), among which 22 encoded AP2 transcription factors, indicating that EtAMA3 deletion has a pronounced effect on the transcriptional regulatory network of *E. tenella*.

In contrast, GO analysis of the upregulated genes showed significant enrichment only in the Cellular Component (CC) category, mainly for membrane-related terms (Fig. 5E, Supplementary material 2). Among the 107 genes associated with these enriched CC terms, 89% encoded proteins with at least one predicted transmembrane domain, and more than half were single-pass transmembrane proteins (Fig. 5F). These findings suggest that EtAMA3 deletion is associated with substantial remodeling of membrane-associated protein expression, which may reflect alterations in parasite surface or parasitophorous vacuole membrane composition.

We also examined the expression of other known components of the moving junction complex. Notably, *RON2*, *RON4*, and *RON8* were significantly downregulated in the EtAMA3-KO line (Fig. 5G), consistent with the notion that loss of EtAMA3 perturbs MJ-associated gene expression and may compromise MJ integrity, thereby contributing to the reduced invasion efficiency observed in EtAMA3-KO sporozoites. In *T. gondii*, the sporozoite-specific TgAMA3 has been shown to interact with the D3 domain of the sporozoite-specific RON2 paralog sporoRON2 (RON2L2), but not with the generic RON2, indicating that TgAMA3 and RON2L2 form a dedicated complex involved in sporozoite invasion [34]. In our study, *RON2L2* was significantly upregulated in the EtAMA3-KO strain (Fig. 5G), which we speculate may represent a compensatory response to EtAMA3 loss. However, the precise role of RON2L2 in *Eimeria* invasion and its functional relationship with EtAMA3 remain to be elucidated.

To verify the reliability of our RNA-Seq transcriptional profiles, we performed RT-qPCR quantification of key MJ genes (RON2, RON4, RON8, RON2L2) and four significantly altered ApiAP2 transcription factors. The RT-qPCR expression trends were consistent with RNA-Seq fold change data (Supplementary material 4), confirming the robustness of our transcriptomic findings.

## Discussion

In this study, we used CRISPR/Cas9-mediated genome editing to characterize the moving junction protein EtAMA3 in *E. tenella* and to dissect its functional contribution to parasite infection. Deletion of EtAMA3 resulted in a moderate but consistent reduction in sporozoite invasion in vitro, together with a marked impairment of parasite replication and oocyst production in vivo, while largely preserving the ability of the parasite to induce protective immunity in chickens. Transcriptomic profiling of the EtAMA3-deficient line further revealed widespread remodeling of gene expression, including altered transcription of multiple MJ components, numerous AP2 transcription factors, and a broad set of membrane-associated proteins, suggesting that EtAMA3 is embedded in a complex network of pathways that collectively regulate parasite invasion and intracellular development.

Our data show that deletion of EtAMA3 led to only an approximately one-third reduction in sporozoite invasion in vitro, yet resulted in a clear attenuation of pathogenicity and a marked decrease in oocyst output in vivo. Notably, transcription of the canonical MJ components *RON2*, *RON4*, and *RON8* was reduced in the EtAMA3-deficient line, whereas *RON2L2* expression was relatively increased. In light of current models in which AMA-RON2/4/8 complexes are essential for maintaining MJ structure and function during apicomplexan invasion [17, 18], these expression changes suggest that loss of EtAMA3 likely perturbs the compositional balance or assembly of the MJ complex and thereby triggers compensatory regulation of MJ-associated genes. In addition, RON2L2 has been reported in *T. gondii* to pair with stage-specific AMA and to participate in sporozoite invasion[34], raising the possibility that *RON2L2* upregulation in the *EtAMA3* knockout represents an attempt by the parasite to recruit alternative RON family members into the MJ to preserve a minimal level of invasive capacity. Consistent with studies in other apicomplexans, AMA proteins are clearly important for invasion, yet individual family members are often not strictly essential, reflecting a degree of functional redundancy or pathway plasticity [17, 21, 35]. The residual invasion observed in the absence of EtAMA3 fits well with this concept of partial redundancy and further implies that, in *E. tenella*, other AMA paralogs or distinct adhesins may partially compensate for EtAMA3 loss. These hypotheses, however, will require formal testing through combinatorial gene knockouts and targeted functional blockade experiments.

Transcriptomic analysis also revealed widespread changes in the expression of AP2 family transcription factors in the EtAMA3 knockout line. ApiAP2 proteins are recognized as key regulators of stage conversion and cell cycle progression in apicomplexan parasites [36, 37]. Because EtAMA3 is a membrane-associated, apically localized protein and is not expected to act directly at the chromatin level, we speculate that the altered AP2 expression profile observed in the AMA3-KO strain is more likely a downstream consequence of perturbations in the invasion machinery or in parasite–host interactions, rather than a direct effect of EtAMA3 on nuclear transcriptional complexes. In light of previous work implicating Ca^2+^ signaling, cGMP/PKG, and multiple CDPK pathways in apicomplexan invasion and development [38–40], it is tempting to speculate that loss of EtAMA3 may influence one or more of these signaling cascades, thereby indirectly reshaping the AP2 regulatory network and its downstream targets. However, the precise signaling axes involved remain to be defined.

Consistent with this apparent remodeling of AP2-mediated regulation, we observed significant enrichment of upregulated genes encoding single-pass transmembrane proteins, many of which harbor predicted transmembrane domains. This pattern suggests that EtAMA3 deletion may be associated with alterations in parasite surface architecture and/or parasitophorous vacuole-associated membranes. One possible explanation is that the parasite attempts to compensate for reduced invasion efficiency and altered host–parasite interface by adjusting the repertoire of surface adhesins, transporters, or receptors.

From both a biological and vaccinological perspective, the EtAMA3 knockout line exhibits a clear attenuated phenotype at conventional infection doses, yet still confers a high level of protection upon challenge. This combination of reduced pathogenicity and preserved immunogenicity aligns well with the conceptual framework of gene deletion live vaccines [41, 42]. It is worth noting, however, that at very high inoculation doses the EtAMA3-KO strain can still induce appreciable cecal lesions, indicating that its virulence, although substantially reduced, is not completely abolished and may benefit from further optimization. Compared with conventional precocious attenuated lines obtained through long-term serial passage, a gene deletion strain offers the advantages of a defined attenuation mechanism and a traceable genetic lesion, features that facilitate quality control and molecular monitoring. In addition, our work further validates the feasibility of using a CRISPR/Cas9-based platform to generate targeted gene knockouts in *E. tenella* [13, 16, 33]. Building on the favorable protective profile of the EtAMA3-KO strain, future studies could introduce additional deletions in genes associated with virulence, gametocyte development, oocyst formation, or key metabolic pathways to explore multigene attenuation strategies, and to systematically evaluate the suitability and performance of such strains as next-generation gene deletion live vaccines for coccidiosis control.

Several considerations should be addressed when interpreting these findings. First, the EtAMA3-KO line represents a highly enriched population containing multiple edited alleles rather than a single cloned knockout, reflecting the technical difficulty of single-oocyst cloning in *Eimeria*. Nevertheless, the reproducibility of the invasion, schizogony, oocyst production, and virulence phenotypes across independent experiments strongly supports a functional association with EtAMA3 disruption. Generation of clonal knockout lines and genetic complementation will be important for defining the precise null phenotype and establishing causality more rigorously. Although complementation was constrained here by the lack of a compatible secondary selectable marker, the stage-specific expression of EtAMA3, the concordant alteration of moving junction-associated genes in sporozoites, and the absence of overt developmental defects following EtAMA3 overexpression provide additional support for the specificity of the observed phenotypes.

The protective efficacy of the EtAMA3-KO line should currently be regarded as proof-of-concept evidence obtained under controlled laboratory conditions. Further development will require systematic evaluation of genetic stability during serial passage, durability of immunity, protection against heterologous field isolates, and safety and efficacy under commercial production conditions. Because the current mutant retains residual virulence at high inoculation doses, additional attenuation or multigene deletion strategies may be required before practical vaccine application.

## Conclusions

In summary, this study demonstrates that EtAMA3 contributes to sporozoite host cell invasion, schizont development, and proliferation of *E. tenella*. Targeted disruption of EtAMA3 reduces parasite virulence and oocyst output while preserving substantial immunoprotective capacity, supporting the EtAMA3-KO line as a preliminary laboratory candidate for genetically attenuated live vaccine development. Transcriptomic analysis further shows that EtAMA3 disruption is associated with altered expression of moving junction-related genes, ApiAP2 transcription factors, and membrane-associated proteins. These changes may reflect compensatory transcriptional remodeling or altered invasion-associated processes, but the underlying mechanisms require further experimental validation. Collectively, these findings advance our understanding of EtAMA3 function and provide proof-of-concept support for its potential application in rational attenuation strategies against chicken coccidiosis.

## Supplementary Information

Supplementary Material 1. Primers used in this study.

Supplementary Material 2. RNA-Seq DEGs and GO enrichments of EtAMA3 knockout strain compared to EtHCYA. Transcriptomic data of *E*. *tenella* from Chen LL *et al*. [33] were employed.

Supplementary Material 3. Statistical analysis data in this study.

Supplementary Material 4. RT-qPCR validation of representative differentially expressed genes. Verification of gene expression by reverse transcription quantitative real-time PCR. The black bars with standard errors indicate fold changes based on the relative expression level determined by qPCR using the 2^−ΔΔCT^ method for three biological replicates. Error bars represent standard deviation (SD).

## Data Availability

The datasets supporting the conclusions of this article are available in the figshare repository [43], https://doi.org/10.6084/m9.figshare.31329073. Raw RNA-Seq sequence read data are available in the NCBI Sequence Read Archive (SRA) repository under the BioProject accession number PRJNA1373537, https://www.ncbi.nlm.nih.gov/bioproject/PRJNA1373537.

## Abbreviations

EtAMA3: *Eimeria tenella* apical membrane antigen 3
*E. tenella*: *Eimeria tenella*
CRISPR-Cas9: Clustered regularly interspaced short palindromic repeats-Cas9
RON2: Rhoptry neck protein 2
RON4: Rhoptry neck protein 4
RON8: Rhoptry neck protein 8
RON2L2: Rhoptry neck protein 2-like 2
AP2: Apicomplexan AP2 transcription factor
MJ: Moving junction
AMA1: Apical membrane antigen 1
AMA2: Apical membrane antigen 2
AMA3: Apical membrane antigen 3
AMA4: Apical membrane antigen 4
EtHCYA: *Eimeria tenella* Houghton line constitutively expressing Cas9
KO: Knockout
OE: Overexpression
gRNA: Guide RNA
sgRNA: Single guide RNA
cDNA: Complementary DNA
UTR: Untranslated Region
DHFR: Dihydrofolate reductase
EYFP: Enhanced yellow fluorescent protein
PCR: Polymerase chain reaction
WT: Wild type
IFA: Indirect immunofluorescence assay
PBS: Phosphate-buffered saline
BSA: Bovine serum albumin
EtM2AP: *Eimeria tenella* microneme-associated protein 2
dpi: Days post-infection
H&E: Hematoxylin and eosin
UUC: Unimmunized-unchallenged control
UCC: Unimmunized-challenged control
ACI: Anticoccidial index
RNA-Seq: RNA sequencing
GO: Gene Ontology
KEGG: Kyoto Encyclopedia of Genes and Genomes
TPM: Transcripts per million
qPCR: Quantitative polymerase chain reaction
ANOVA: Analysis of variance
BP: Biological process
MF: Molecular function
CC: Cellular component
DEGs: Differentially expressed genes

## References

[1] Dubey JP. Coccidiosis in livestock, poultry, companion animals, and humans. London: CRC Press; 2019.

[2] Williams RB. A compartmentalised model for the estimation of the cost of coccidiosis to the world’s chicken production industry. Int J Parasitol. 1999;29:1209–29. 10.1016/s0020-7519(99)00086-7 10576573

[3] Blake DP, Knox J, Dehaeck B, Huntington B, Rathinam T, Ravipati V, et al. Re-calculating the cost of coccidiosis in chickens. Vet Res. 2020;51:1–14. 10.1186/s13567-020-00837-2 PMC7488756 32928271

[4] Blake DP, Tomley FM. Securing poultry production from the ever-present *Eimeria* challenge. Trends Parasitol. 2014;30:12–9. 10.1016/j.pt.2013.10.003 24238797

[5] Clark EL, Tomley FM, Blake DP. Are *Eimeria* genetically diverse, and does it matter? Trends Parasitol. 2017;33:231–41. 10.1016/j.pt.2016.08.007 27593338

[6] Chapman HD, Cherry TE, Danforth HD, Richards G, Shirley MW, Williams RB. Sustainable coccidiosis control in poultry production: the role of live vaccines. Int J Parasitol. 2002;32:617–29. 10.1016/s0020-7519(01)00362-9 11943233

[7] Peek H, Landman W. Coccidiosis in poultry: anticoccidial products, vaccines and other prevention strategies. Vet Q. 2011;31:143–61. 10.1080/01652176.2011.605247 22029884

[8] Meeusen EN, Walker J, Peters A, Pastoret P-P, Jungersen G. Current status of veterinary vaccines. Clin Microbiol Rev. 2007;20:489–510. 10.1128/CMR.00005-07 PMC1932753 17630337

[9] Sánchez-Valdéz FJ, Pérez Brandán C, Ferreira A, Basombrío MÁ. Gene-deleted live-attenuated *Trypanosoma cruzi* parasites as vaccines to protect against Chagas disease. Expert Rev Vaccines. 2015;14:681–97. 10.1586/14760584.2015.989989 25496192

[10] Fox BA, Bzik DJ. Nonreplicating, cyst-defective type II *Toxoplasma gondii* vaccine strains stimulate protective immunity against acute and chronic infection. Infect Immun. 2015;83:2148–55. 10.1128/IAI.02756-14 PMC4399033 25776745

[11] Zhang N-Z, Chen J, Wang M, Petersen E, Zhu X-Q. Vaccines against *Toxoplasma gondii*: new developments and perspectives. Expert Rev Vaccines. 2013;12:1287–99. 10.1586/14760584.2013.844652 24093877

[12] Vaughan A, Wang R, Kappe SH. Genetically engineered, attenuated whole-cell vaccine approaches for malaria. Hum Vaccin Immunother. 2010;6:107–13. 10.4161/hv.6.1.9654 PMC3641786 19838068

[13] Hu D, Tang X, Ben Mamoun C, Wang C, Wang S, Gu X, et al. Efficient single-gene and gene family editing in the Apicomplexan parasite *Eimeria tenella* using CRISPR-Cas9. Front Bioeng Biotechnol. 2020;8:128. 10.3389/fbioe.2020.00128 PMC7052334 32158750

[14] Tang X, Suo J, Liang L, Duan C, Hu D, Gu X, et al. Genetic modification of the protozoan *Eimeria tenella* using the CRISPR/Cas9 system. Vet Res. 2020;51:1–5. 10.1186/s13567-020-00766-0 PMC7065449 32160917

[15] Cheng P, Zhang Z, Yang F, Cai S, Wang L, Wang C, et al. FnCas12a/crRNA-Mediated genome editing in *Eimeria tenella*. Front Genet. 2021;12:738746. 10.3389/fgene.2021.738746 PMC8494306 34630528

[16] Li P, Su R, Ren G, Si H, Song X, Hu D. Molecular mechanisms of surface antigen suppression by ApiAP2 and its implications for vaccine development. Vet Res. 2025;56:63. 10.1186/s13567-025-01491-2 PMC11929185 40121506

[17] Besteiro S, Dubremetz JF, Lebrun M. The moving junction of apicomplexan parasites: a key structure for invasion. Cell Microbiol. 2011;13:797–805. 10.1111/j.1462-5822.2011.01597.x 21535344

[18] Shen B, Sibley LD. The moving junction, a key portal to host cell invasion by apicomplexan parasites. Curr Opin Microbiol. 2012;15:449–55. 10.1016/j.mib.2012.02.007 PMC3387501 22445360

[19] Straub KW, Peng ED, Hajagos BE, Tyler JS, Bradley PJ. The moving junction protein RON8 facilitates firm attachment and host cell invasion in *Toxoplasma gondii*. PLoS Pathog. 2011;7:e1002007. 10.1371/journal.ppat.1002007 PMC3053350 21423671

[20] Beck JR, Chen AL, Kim EW, Bradley PJ. RON5 is critical for organization and function of the *Toxoplasma* moving junction complex. PLoS Pathog. 2014;10:e1004025. 10.1371/journal.ppat.1004025 PMC3961375 24651769

[21] Lamarque MH, Roques M, Kong-Hap M, Tonkin ML, Rugarabamu G, Marq J-B, et al. Plasticity and redundancy among AMA–RON pairs ensure host cell entry of *Toxoplasma* parasites. Nat Commun. 2014;5:4098. 10.1038/ncomms5098 24934579

[22] Eckert J, Braun R, Shirley MW, Coudert P. Biotechnology: guidelines on techniques in coccidiosis research. Luxembourg: European Commission Directorate-General XII, Science, Research and Development; 1995.

[23] Duan C, Tang X, Hu D, Zhang S, Liu J, Bi F, et al. Nucleofection and in vivo propagation of chicken *Eimeria* parasites. J Vis Exp. 2020;156:e60552. 10.3791/60552. 32116306

[24] Johnson J, Reid WM. Anticoccidial drugs: lesion scoring techniques in battery and floor-pen experiments with chickens. Exp Parasitol. 1970;28:30–6. 10.1016/0014-4894(70)90063-9 5459870

[25] Zhang L, Song Z, Li P, Song X, Tang X, Hu D. Molecular characterization and immune protective efficacy of 3 *Eimeria tenella* antigens. Poult Sci. 2024;103:103234. 10.1016/j.psj.2023.103234 PMC10685023 37980744

[26] Aunin E, Böhme U, Blake D, Dove A, Smith M, Corton C, et al. The complete genome sequence of *Eimeria tenella* (Tyzzer 1929), a common gut parasite of chickens. Wellcome Open Res. 2021;6:225. 10.12688/wellcomeopenres.17100.1 PMC8515493 34703904

[27] Kim D, Paggi JM, Park C, Bennett C, Salzberg SL. Graph-based genome alignment and genotyping with HISAT2 and HISAT-genotype. Nat Biotechnol. 2019;37:907–15. 10.1038/s41587-019-0201-4 PMC7605509 31375807

[28] Li H, Handsaker B, Wysoker A, Fennell T, Ruan J, Homer N, et al. The sequence alignment/map format and SAMtools. Bioinformatics. 2009;25:2078–9. 10.1093/bioinformatics/btp352 PMC2723002 19505943

[29] Anders S, Pyl PT, Huber W. HTSeq—a Python framework to work with high-throughput sequencing data. Bioinformatics. 2015;31:166–9. 10.1093/bioinformatics/btu638 PMC4287950 25260700

[30] Love MI, Huber W, Anders S. Moderated estimation of fold change and dispersion for RNA-seq data with DESeq2. Genome Biol. 2014;15:550. 10.1186/s13059-014-0550-8 PMC4302049 25516281

[31] Wu T, Hu E, Xu S, Chen M, Guo P, Dai Z, et al. ClusterProfiler 4.0: a universal enrichment tool for interpreting omics data. Innovation. 2021;2:100141. 10.1016/j.xinn.2021.100141 PMC8454663 34557778

[32] Wang Q, Zhu S, Zhao Q, Huang B, Yu S, Yu Y, et al. Identification and characterization of a novel apical membrane antigen 3 in *Eimeria tenella*. J Eukaryot Microbiol. 2021;68:e12836. 10.1111/jeu.12836 33289220

[33] Chen L, Tang X, Sun P, Hu D, Zhang Y, Wang C, et al. Comparative transcriptome profiling of *Eimeria tenella* in various developmental stages and functional analysis of an ApiAP2 transcription factor exclusively expressed during sporogony. Parasit Vectors. 2023;16:241. 10.1186/s13071-023-05828-8 PMC10354945 37468981

[34] Poukchanski A, Fritz HM, Tonkin ML, Treeck M, Boulanger MJ, Boothroyd JC. *Toxoplasma gondii* sporozoites invade host cells using two novel paralogues of RON2 and AMA1. PLoS ONE. 2013;8:e70637. 10.1371/journal.pone.0070637 PMC3734201 23940612

[35] Bargieri DY, Andenmatten N, Lagal V, Thiberge S, Whitelaw JA, Tardieux I, et al. Apical membrane antigen 1 mediates apicomplexan parasite attachment but is dispensable for host cell invasion. Nat Commun. 2013;4:2552. 10.1038/ncomms3552 PMC3826631 24108241

[36] Jeninga MD, Quinn JE, Petter M. ApiAP2 transcription factors in apicomplexan parasites. Pathogens. 2019;8:47. 10.3390/pathogens8020047 PMC6631176 30959972

[37] Zarringhalam K, Ye S, Lou J, Rezvani Y, Gubbels M-J. Cell cycle-regulated ApiAP2s and parasite development: the *Toxoplasma* paradigm. Curr Opin Microbiol. 2023;76:102383. 10.1016/j.mib.2023.102383 PMC10840917 37898053

[38] Billker O, Lourido S, Sibley LD. Calcium-dependent signaling and kinases in apicomplexan parasites. Cell Host Microbe. 2009;5:612–22. 10.1016/j.chom.2009.05.017 PMC2718762 19527888

[39] Nagamune K, Moreno SN, Chini EN, Sibley LD. Calcium regulation and signaling in apicomplexan parasites. In: Burleigh BA, Soldati-Favre D, editors. Molecular mechanisms of parasite invasion. New York: Springer; 2008. p. 70–81. 10.1007/978-0-387-78267-6_5 18512342

[40] Uboldi AD, Wilde ML, Bader SM, Tonkin C. Environmental sensing and regulation of motility in Toxoplasma. Mol Microbiol. 2021;115:916–29. 10.1111/mmi.14661 33278047

[41] Gao Y, Sun P, Hu D, Tang X, Zhang S, Shi F, et al. Advancements in understanding chicken coccidiosis: from *Eimeria* biology to innovative control strategies. One Health Adv. 2024;2:6.

[42] Liu Q, Liu X, Zhao X, Zhu X-Q, Suo X. Live attenuated anticoccidial vaccines for chickens. Trends Parasitol. 2023;39:1087–99. 10.1016/j.pt.2023.09.002 37770352

[43] Du S, Pang Y, Dong R, Tang X, Ou C, Si H, et al. Functional characterization of EtAMA3 in *Eimeria tenella* and immunogenicity of its knockout line data sets. figshare. 2026. 10.6084/m9.figshare.31329073. 10.1186/s13071-026-07618-4 PMC13760855 43021396

