# Supplementary material for: Functional characterization of EtAMA3 in Eimeria tenella and immunogenicity of its knockout line
Source: Parasit Vectors. 2026 Aug 19;19:493. doi: 10.1186/s13071-026-07618-4 (PMC13760855; doi:10.1186/s13071-026-07618-4)
Supplement: Supplementary file 1 [file 13071_2026_7618_MOESM4_ESM.pdf]

Supplementary Figure

Supplementary Figure 1

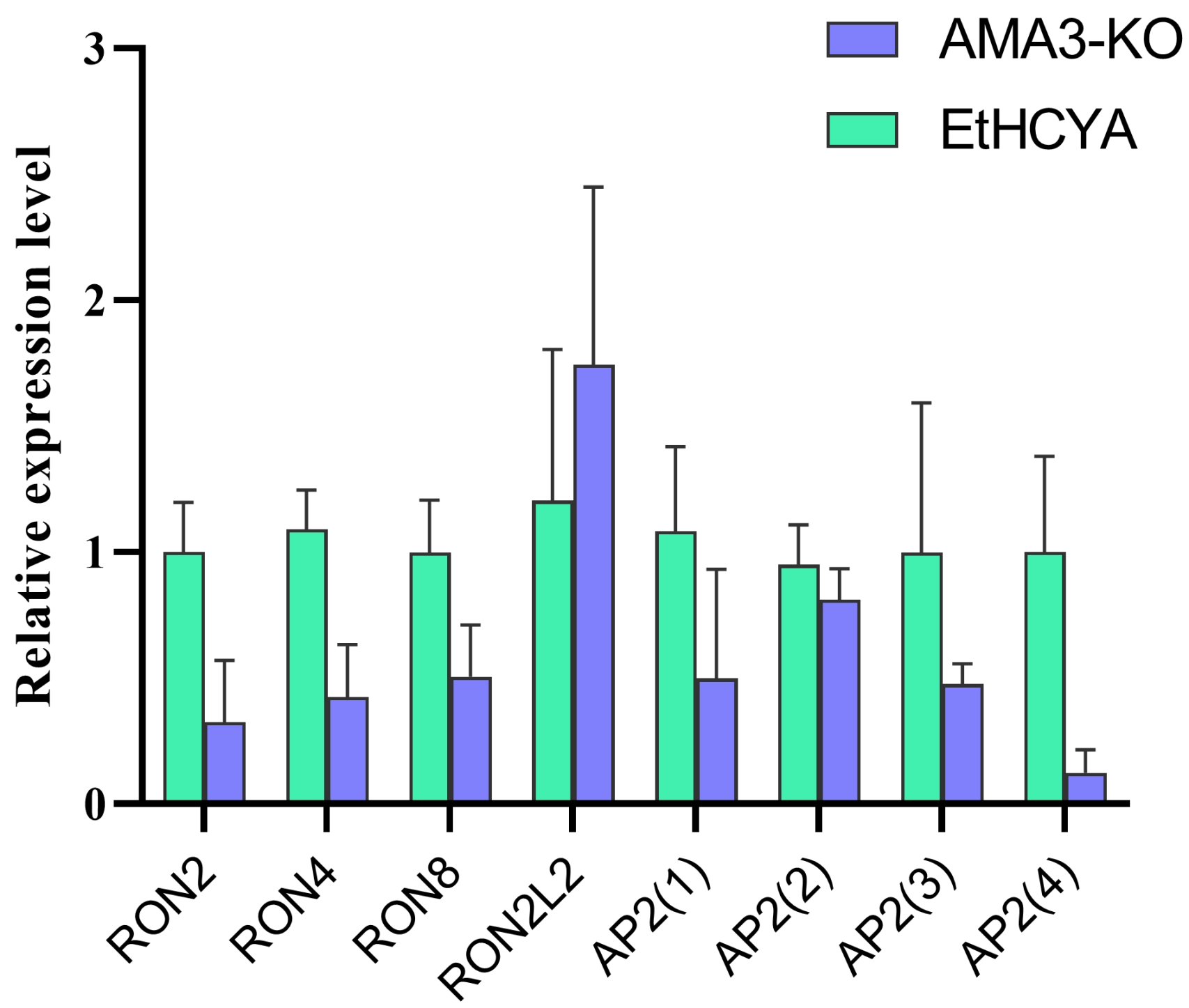

RT- qPCR validation of representative differentially expressed genes.
